# Heart failure hospitalizations and clinical outcomes in patients undergoing tricuspid transcatheter edge‐to‐edge repair: Insights from EuroTR

**DOI:** 10.1002/ejhf.3757

**Published:** 2025-07-18

**Authors:** Daniela Tomasoni, Marianna Adamo, Jörg Hausleiter, Elisa Pezzola, Karl‐Patrik Kresoja, Jennifer von Stein, Vera Fortmeier, Christoph Pauschinger, Wolfgang Rottbauer, Mohammad Kassar, Bjoern Goebel, Paolo Denti, Paul Achouh, Tienush Rassaf, Manuel Barreiro‐Perez, Peter Boekstegers, Andreas Rück, Monika Zdanyte, Flavien Vincent, Philipp Schlegel, Ralph‐Stephan von Bardeleben, Mirjam G. Wild, Christian Besler, Stephanie Brunner, Stefan Toggweiler, Julia Grapsa, Tiffany Patterson, Holger Thiele, Tobias Kister, Giuseppe Tarantini, Giulia Masiero, Marco De Carlo, Alessandro Sticchi, Mathias H. Konstandin, Eric Van Belle, Tobias Geisler, Rodrigo Estévez‐Loureiro, Peter Luedike, Nicole Karam, Francesco Maisano, Philipp Lauten, Fabien Praz, Mirjam Kessler, Daniel Kalbacher, Volker Rudolph, Christos Iliadis, Philipp Lurz, Lukas Stolz, Marco Metra, the EuroTR Investigators, Edoardo Pancaldi, Luca Branca, Ludwig T. Weckbach, Julia Novotny, Roman Pfister, Stephan Baldus, Muhammed Gerçek, Felix Rudolph, Sebastian Ludwig, Benedikt Koell, Leonhard‐Moritz Schneider, Dominik Felbel, Carsten Salomon, Harald Lapp, Tania Puscas, Alain Berrebi, Amir Abbas Mahabadi, Florian Schindhelm, Berenice Caneiro‐Queija, Julio C. Echarte, Jürgen Schreieck, Andreas Goldschmied, Natacha Rousse, Samy Aghezzaf, Norbert Frey, Martin J. Kraus, Leonie Ziegler, Sebastian Rosch, Federico Arturi, Andrea Panza, Matteo Mazzola, Cristina Giannini

**Affiliations:** ^1^ Cardiology. ASST Spedali Civili di Brescia and Department of Medical and Surgical Specialties, Radiological Sciences, and Public Health University of Brescia Brescia Italy; ^2^ Department of Clinical Science and Education, Södersjukhuset Karolinska Institutet Stockholm Sweden; ^3^ Medizinische Klinik und Poliklinik I, LMU Klinikum, LMU München Munich Germany; ^4^ German Center for Cardiovascular Research (DZHK), partner site Munich Heart Alliance Munich Germany; ^5^ Department of Cardiology, Cardiology I University Medical Center of the Johannes Gutenberg‐University Mainz Mainz Germany; ^6^ Department of Cardiology Heart Center, University of Cologne Cologne Germany; ^7^ Department of General and Interventional Cardiology Heart and Diabetes Center North Rhine‐Westphalia, Ruhr University Bochum Bad Oeynhausen Germany; ^8^ Department of Cardiology University Heart & Vascular Center Hamburg, University Medical Center Hamburg‐Eppendorf Hamburg Germany; ^9^ German Center of Cardiovascular Research (DZHK), partner site Hamburg/Kiel/Lübeck Hamburg Germany; ^10^ Department of Cardiology University Heart Center Ulm Ulm Germany; ^11^ Department of Cardiology Inselspital Bern, Bern University Hospital Bern Switzerland; ^12^ Department of Cardiology Heart Center, Zentralklinik Bad Berka Bad Berka Germany; ^13^ Heart Valve Center, Cardio‐Thoracic‐Vascular Department IRCCS Milan Italy; ^14^ Cardiology Department European Hospital Georges Pompidou, Université Cité Paris France; ^15^ Department of Cardiology and Vascular Medicine University Hospital Essen, University Duisburg‐Essen, West German Heart and Vascular Center Essen Germany; ^16^ Division of Interventional Cardiology Hospital Álvaro Cunqueiro Vigo Spain; ^17^ Department of Cardiology Helios Klinikum Siegburg Siegburg Germany; ^18^ Department of Cardiology Karolinska University Hospital Stockholm Sweden; ^19^ Medical Clinic III, University Hospital Tübingen Tübingen Germany; ^20^ Cardiology Department Centre Hospitalier Universitaire De Lille Lille France; ^21^ Division of Cardiology, Department of Internal Medicine III University Hospital Heidelberg, Ruprecht‐Karl University Heidelberg Heidelberg Germany; ^22^ University Heart Center Freiburg/Bad Krozingen Bad Krozingen Germany; ^23^ Heart Center Lucerne, Luzerner Kantonsspital Lucerne Switzerland; ^24^ Department of Cardiology Guys and St Thomas NHS Trust London UK; ^25^ Department of Cardiology Heart Center Leipzig at Leipzig University Leipzig Germany; ^26^ Department of Cardiac, Thoracic Vascular Sciences and Public Health University of Padua Padua Italy; ^27^ Cardiothoracic and Vascular Department Azienda Ospedaliero‐Universitaria Pisana Pisa Italy

**Keywords:** Heart failure hospitalization, Tricuspid regurgitation, Transcatheter edge‐to‐edge repair, T‐TEER, Mortality

## Abstract

**Aims:**

To assess the prevalence, prognostic significance, and predictors of heart failure hospitalization (HFH) before and after tricuspid transcatheter edge‐to‐edge repair (T‐TEER) in a large real‐world cohort of patients with tricuspid regurgitation (TR).

**Methods and results:**

Data from the European Registry of Transcatheter Repair for Tricuspid Regurgitation (EuroTR registry) were analysed. Among 1000 patients undergoing T‐TEER for symptomatic TR, 361 (36.1%) had no HFH, 459 (45.9%) had one single HFH, and 180 (18.0%) had multiple HFH the year before T‐TEER. Patients with any HFH had more severe heart failure compared with those without. Procedural success (residual TR ≤2) did not differ between patients with single, multiple, or no HFHs before T‐TEER. Multivariable analysis showed that a history of HFH was associated with an increased mortality risk (adjusted hazard ratio [HR] 1.51, 95% confidence interval [CI] 1.11–2.06 for single vs. no HFH; adjusted HR 1.63, 95% CI 1.15–2.31 for multiple vs. no HFH), and a higher risk of the combined endpoint of all‐cause mortality or HFH. HFH risk decreased by 72% in the 1 year following T‐TEER compared to the previous year. Procedural success was the sole independent predictor for reducing HFHs.

**Conclusions:**

In the EuroTR cohort, a history of HFH was highly prevalent and associated with worse clinical outcomes. Among high‐risk patients with symptomatic TR, T‐TEER significantly lowered HFH risk, with residual TR grade ≤2 being the key predictor for reduced HFH incidence.

## Introduction

A history of heart failure hospitalization (HFH) is a well‐recognized prognostic marker in patients with left‐sided heart failure (HF), strongly linked to an increased risk of readmissions, all‐cause mortality, and cardiovascular (CV) death.^1^, ^2^, ^3^, ^4^, ^5^, ^6^, ^7^ Moderate or severe tricuspid regurgitation (TR) is prevalent among HF patients, with rates ranging between 10% and 39%.^8^, ^9^, ^10^, ^11^, ^12^, ^13^, ^14^, ^15^ TR is associated with a higher risk of residual congestion at discharge, HF readmissions, and mortality.^9^, ^13^, ^16^, ^17^, ^18^, ^19^

Emerging treatments, such as tricuspid transcatheter edge‐to‐edge repair (T‐TEER), offer new therapeutic options for patients with relevant TR. The TRILUMINATE Pivotal trial (Trial to Evaluate Cardiovascular Outcomes in Patients Treated with the Tricuspid Valve Repair System Pivotal) demonstrated that T‐TEER effectively reduces TR severity and improves quality of life up to 1 year post‐intervention compared to medical therapy, though it showed no significant between‐group differences in mortality or HFH at 1‐year follow‐up.^20^

However, only 25% of patients in this trial had a history of HFH, leaving the impact of T‐TEER on HFH reduction uncertain.

The prevalence of HFH in patients undergoing T‐TEER and its association with clinical outcomes remains largely unexplored. This study aimed to assess the prevalence and prognostic impact of HFH in the year prior to T‐TEER, as well as changes in HFH rates after the procedure and the predictors of these outcomes, in a large real‐world cohort of patients treated for symptomatic TR.

## Methods

### Study population

The multicentre, real‐world, observational, European Registry of Transcatheter Repair for Tricuspid Regurgitation (EuroTR registry) included patients who underwent T‐TEER for symptomatic TR between March 2016 and February 2024 at 24 European centres. Treatment decisions were made by an interdisciplinary Heart Team, comprising specialists in HF, cardiac surgery, CV imaging, and interventional cardiology in accordance with international guidelines.^21^ Prior to the procedure, all patients remained symptomatic despite receiving maximum tolerated dosages of diuretic medications. T‐TEER was performed using either the PASCAL device (Edwards Lifesciences, Irvine, CA, USA) or the TriClip system (Abbott, Santa Clara, CA, USA).

Patients with incomplete data on HFH were excluded from this analysis. Baseline clinical and echocardiographic data were retrospectively collected, and follow‐up assessments were conducted via clinical visits and/or phone consultations.

This study adheres to the principles of the Declaration of Helsinki, was approved by the Ethics Committee, and is registered in ClinicalTrials.gov (NCT06307262).

### Definitions and outcomes

Patients were stratified into three groups based on the number of HFHs in the year preceding T‐TEER: (1) no HFH; (2) single HFH (one event); (3) multiple HFHs (more than one event).

Post T‐TEER trajectories in HFH were defined based on the occurrence of HFH in the year before and after the procedure: (1) ‘improved’ – HFH occurring before but not after T‐TEER; (2) ‘stable’ – HFH occurring both before and after, or not occurring in either period; (3) ‘worsened’ – HFH occurring only after T‐TEER, or if the patient died within 1 year post T‐TEER, regardless of HFHs. The ‘stable’ and ‘worsened’ groups were combined to form a dichotomous variable: ‘improved’ versus ‘not improved’ (i.e. stable or worsened).

Tricuspid regurgitation severity was graded on a five‐level scale: mild (grade 1), moderate (grade 2), severe (grade 3), massive (grade 4), and torrential (grade 5).^22^ Residual TR was evaluated at discharge following T‐TEER.

The primary outcome was all‐cause mortality. Additionally, the composite endpoint of all‐cause death or HFH was assessed.

### Statistical analysis

Baseline characteristics are presented by categories defined according to the number of HFHs in the 1 year prior to T‐TEER (no HFH vs. single HFH vs. multiple HFHs).

Categorical variables are presented as number and percentage and tested for differences between groups with the *χ* ^2^ test. Continuous variables are presented with median and interquartile range and tested for differences between groups with the Kruskal–Wallis test.

The first occurrence of all‐cause mortality and of the composite endpoint was evaluated in patients with single and multiple HFHs versus those not hospitalized using the Kaplan–Meier method and the log‐rank test. Multivariable Cox proportional hazards models were performed to assess the association between HFH and outcomes, including as covariates the variables associated with the outcome at univariable analysis (with a *p* < 0.001) and/or deemed relevant: age, sex, New York Heart Association (NYHA) class, haemoglobin, estimated glomerular filtration rate (eGFR), gamma‐glutamyl transferase (GGT), left ventricular ejection fraction (LVEF), tricuspid annular plane systolic excursion (TAPSE) and residual TR. Additional sensitivity analyses were performed including into the main model additional variables potentially associated with outcomes (online supplementary material).

Results of the Cox regression analyses were reported as hazard ratio (HR) and 95% confidence interval (CI). Multicollinearity was assessed using the variance inflation factor.

Differences in the risk of hospitalizations before and after T‐TEER were calculated and the relative risk ratio (RR) was presented with 95% CI. Predictors of improvement in HFH status after T‐TEER were evaluated using logistic regression models, partly crude, partly adjusting for variables shown in the forest plot (i.e. age, sex, NYHA class, haemoglobin, eGFR, GGT, LVEF, TAPSE, daily furosemide‐equivalent doses, and residual TR). Variables were selected based on clinical relevance and significance at crude analysis.

A sensitivity analysis was performed excluding patients with haemodynamic significant mitral regurgitation undergoing mitral TEER (M‐TEER) within the study period (*n* = 159).

All analyses were performed using STATA version 16.1 (Stata Corp., College Station, TX, USA). The level of significance was set to 5%, two‐sided.

## Results

Overall, 2152 patients were enrolled in the EuroTR registry. For the purpose of the present analysis, patients with missing data regarding the number of HFH prior to T‐TEER (*n* = 1152) were excluded. Thus, 1000 patients undergoing T‐TEER (57% receiving TriClip and 43% the PASCAL device) were finally included. Baseline characteristics of the population included versus excluded are reported in (online supplementary *Table* *S1*). Patients excluded from the analysis seems to have a slightly lower risk of mortality as compared to the included population (1‐year mortality rate: 17 [14–20] per 100 patients/year vs. 24 [20–27] per 100 patients/year).

Among patients included, 361 (36.1%) had no HFH during the 1 year before T‐TEER and 639 (63.9%) had at least one HFH, with 459 (45.9%) experiencing a single HFH and 180 (18.0%) multiple HFHs, respectively.

### Baseline characteristics and procedural data according to heart failure hospitalization history

**Figure 1:**
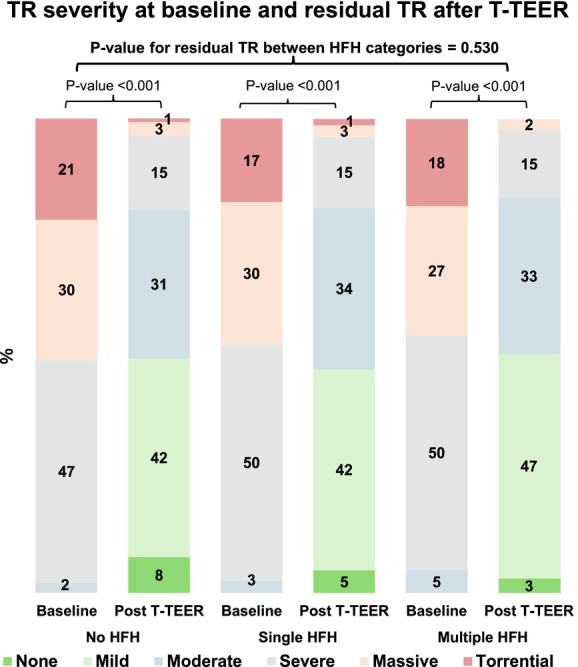
Tricuspid regurgitation (TR) severity at baseline and residual TR after tricuspid transcatheter edge‐to‐edge repair (T‐TEER), stratified by heart failure hospitalization (HFH) categories.

Patients with at least a single HFH, compared to those without, had more severe symptoms and signs of HF, more impaired exercise capacity and received higher doses of diuretics (*Table* *1*). They also had worse renal function, higher N‐terminal pro‐B‐type natriuretic peptide (NT‐proBNP) concentrations, greater left atrium and right chambers dilatations despite similar distribution of TR grades, as well as similar left and right ventricular systolic function (*Table* *2*). Post‐procedural residual TR was not different among the three groups (*Figure* *1*), with 291 (81%), 372 (81%) and 150 (83%) achieving residual TR ≤2 among patients with no HFH, single and multiple HFHs, respectively. Baseline characteristics and post‐procedural residual TR of the study population after the exclusion of patients undergoing M‐TEER are shown in online supplementary *Table* *S2*.

**Table 1:** Baseline clinical characteristics of the study population stratified by the number of heart failure hospitalizations

Variable	No HFH (*n* = 361, 36%)	Single HFH (*n* = 459, 46%)	Multiple HFH (*n* = 180, 18%)	*p*‐value
Age (years)	80 (76–83)	80 (77–84)	79 (74–83)	0.006
Female sex	193 (53.5)	246 (53.6)	73 (40.6)	0.007
BMI (kg/m^2^)	25 (23–29)	25 (22–28)	24 (23–28)	0.15
Arterial hypertension	297 (82.3)	388 (84.5)	158 (87.8)	0.25
Dyslipidaemia	133 (37.0)	165 (36.1)	79 (44.1)	0.16
Diabetes mellitus	72 (19.9)	112 (24.4)	54 (30.0)	0.03
Previous myocardial infarction	34 (9.4)	52 (11.3)	27 (15.0)	0.15
Coronary artery disease	173 (47.9)	220 (47.9)	89 (49.4)	0.93
Peripheral artery disease	40 (14.1)	50 (18.2)	15 (23.8)	0.14
Previous stroke/TIA	38 (10.5)	56 (12.2)	21 (11.7)	0.75
Atrial fibrillation/flutter	335 (92.8)	415 (90.4)	167 (92.8)	0.40
COPD	44 (12.2)	87 (19.0)	46 (25.6)	<0.001
History of cardiac surgery	94 (26.0)	104 (22.7)	52 (28.9)	0.22
Prior TV surgery	5 (1.8)	3 (0.7)	0 (0.0)	0.12
RV lead	104 (28.8)	134 (29.2)	61 (33.9)	0.43
NYHA class				
I	5 (1.4)	3 (0.7)	0 (0.0)	<0.001
II	49 (13.6)	32 (7.0)	12 (6.7)	
III	270 (74.8)	332 (72.5)	128 (1.5)	
IV	37 (10.2)	91 (19.9)	39 (21.8)	
6MWT (m)	246 (175–325)	200 (132–300)	220 (140–296)	0.001
TRI‐SCORE	6 (5–7)	6 (5–8)	7 (5–8)	<0.001
Predicted in‐hospital mortality^a^	22 (14–34)	22 (14–48)	32 (18–33)	<0.001
Heart rate (bpm)	71 (63–82)	72 (63–82)	74 (66–85)	0.19
Peripheral oedema	191 (52.9)	324 (70.7)	145 (80.6)	<0.001
Ascites	17 (4.7)	76 (16.6)	44 (24.4)	<0.001
Pleural effusion	41 (11.7)	116 (26.0)	74 (42.0)	<0.001
Any sign of RHF	233 (66.2)	365 (80.4)	157 (87.2)	<0.001
Loop diuretic	325 (91.0)	429 (93.9)	172 (96.1)	0.069
Furosemide‐equivalent daily dose (mg)	40 (20–80)	40 (20–80)	60 (40–120)	<0.001
Thiazide diuretic	54 (15.0)	106 (23.1)	41 (22.9)	0.009
MRA	135 (37.4)	198 (43.2)	87 (48.6)	0.036
Beta‐blockers	299 (83.1)	400 (87.3)	157 (87.7)	0.16
RASI	220 (60.9)	280 (61.3)	112 (62.9)	0.90
SGLT2‐i	35 (10.1)	54 (12.2)	19 (11.9)	0.63

Categorical variables are presented as *n* (%), continuous variables are presented as median (interquartile range).

6MWT, 6‐min walking test; BMI, body mass index; COPD, chronic obstructive pulmonary disease; HFH, heart failure hospitalization; MRA, mineralocorticoid receptor antagonist; NYHA, New York Heart Association; RASI, renin–angiotensin system inhibitor; RHF, right heart failure; RV, right ventricular; SGLT2‐i, sodium–glucose co‐transporter 2 inhibitor; STS, Society of Thoracic Surgeons; TIA, transient ischaemic attack; TV, tricuspid valve.

^a^ Predicted in‐hospital mortality according to the TRI‐SCORE value.

**Table 2:** Baseline laboratory, right heart catheterization and echocardiographic characteristics of the study population stratified by the number of heart failure hospitalizations

Variables	No HFH (*n* = 361, 36%)	Single HFH (*n* = 459, 46%)	Multiple HFH (*n* = 180, 18%)	*p*‐value
**Laboratory**				
Haemoglobin (g/dl)	12.1 (10.7–13.2)	11.6 (10.2–12.9)	11.2 (9.6–12.4)	<0.001
Platelet count (*1000/L)	186 (150–228)	189 (152–234)	182 (155–277)	0.82
NT‐proBNP (pg/L)	2195 (1180–4008)	3009 (1803–5608)	3138 (1702–6823)	<0.001
Creatinine (mg/dl)	1.22 (0.99–1.64)	1.40 (1.06–1.80)	1.42 (1.10–1.96)	<0.001
eGFR (ml/min)	49 (33–67)	44 (33–58)	43 (29–56)	0.002
Total bilirubin (mg/dl)	0.88 (0.60–1.16)	0.80 (0.59–1.20)	0.82 (0.53–1.23)	1.00
AST (U/L)	29 (24–36)	28 (23–36)	27 (22–36)	0.47
ALT (U/L)	19 (14–26)	18 (13–24)	17 (13–24)	0.12
GGT (U/L)	82 (45–161)	97 (52–177)	122 (57–222)	0.002
Alkaline phosphatase (U/L)	94 (69–128)	102 (78–139)	102 (66–129)	0.37
INR	1.2 (1.2–1.6)	1.3 (1.1–1.7)	1.3 (1.1–1.7)	0.24
**Echocardiography**				
LVEF (%)	55 (47–60)	55 (47–60)	54 (43–60)	0.41
LA volume (ml)	91 (64–125)	104 (75–145)	117 (90–159)	<0.001
MR severity				
No‐trace	22 (6.2)	25 (5.6)	11 (6.1)	0.005
Mild	194 (54.5)	245 (54.6)	105 (58.3)	
Moderate	109 (30.6)	104 (23.1)	29 (16.1)	
Moderate‐to‐severe	24 (6.7)	61 (13.6)	26 (14.4)	
Severe	7 (2.0)	14 (3.1)	9 (5.0)	
RV basal EDD (mm)	47 (42–54)	48 (43–54)	49 (43–55)	0.12
RV mid EDD (mm)	38 (32–44)	40 (34–45)	41 (36–47)	<0.001
RV EDA (cm^2^)	23 (18–30)	25 (20–32)	29 (24–35)	<0.001
RV FAC	38 (30–44)	38 (31–43)	37 (29–41)	0.27
TAPSE (mm)	17 (14–20)	17 (14–20)	17 (13–19)	0.19
TR severity				
Mild	1 (0.3)	0 (0.0)	0 (0.0)	0.45
Moderate	7 (2.0)	13 (2.8)	9 (5.0)	
Severe	168 (46.9)	227 (49.6)	89 (49.4)	
Massive	106 (29.6)	138 (30.1)	49 (27.2)	
Torrential	76 (21.2)	80 (17.5)	33 (18.3)	
TR aetiology				
Primary	11 (3.1)	22 (4.8)	15 (8.3)	0.11
Secondary	317 (89.0)	394 (86.6)	149 (82.8)	
Mixed	28 (7.9)	39 (8.6)	16 (8.9)	
TR EROA (cm^2^)	0.59 (0.41–0.86)	0.56 (0.40–0.80)	0.48 (0.35–0.73)	0.002
TR regurgitant volume (ml)	47 (36–64)	46 (36–64)	42 (30–58)	0.002
Coaptation gap (mm)	6 (4–7)	5 (4–8)	5 (4–7)	0.60
RA area (cm^2^)	32 (28–41)	34 (28–44)	35 (29–43)	0.023
Echo‐PASP (mmHg)	37 (30–49)	41 (32–50)	42 (32–52)	0.019
**Right heart catheterization**				
RAP mean (mmHg)	13 (9–17)	13 (9–17)	13 (10–17)	0.84
RA V‐wave (mmHg)	17 (11–22)	17 (11–23)	18 (13–24)	0.64
PAP systolic (mmHg)	42 (34–52)	45 (36–56)	50 (41–60)	<0.001
PAP diastolic (mmHg)	17 (13–22)	19 (15–24)	23 (17–28)	<0.001
PAP mean (mmHg)	27 (21–34)	30 (23–36)	32 (27–40)	<0.001
PCWP mean (mmHg)	17 (12–22)	19 (14–24)	21 (14–26)	<0.001
CO (L/min)	4 (3–5)	4 (3–5)	5 (4–6)	<0.001
PVR	2 (2–4)	3 (2–4)	2 (2–4)	0.44

Categorical variables are presented as *n* (%), continuous variables are presented as median (interquartile range).

ALT, alanine transaminase; AST, aspartate transaminase; CO, cardiac output; EDA, end‐diastolic area; EDD, end‐diastolic diameter; eGFR, estimated glomerular filtration rate; EROA, effective regurgitant orifice area; FAC, fractional area change; GGT, gamma‐glutamyl transferase; HFH, heart failure hospitalization; INR, international normalized ratio; LA, left atrial; LVEF, left ventricular ejection fraction; MR, mitral regurgitation; NT‐proBNP, N‐terminal pro‐B‐type natriuretic peptide; PAP, pulmonary artery pressure; PCWP, pulmonary capillary wedge pressure; PASP, pulmonary artery systolic pressure; PVR, pulmonary vascular resistance; RA, right atrial; RAP, right arial pressure; RV, right ventricular; TAPSE, tricuspid annular plane systolic excursion; TR, tricuspid regurgitation; TV, tricuspid valve.

### Outcomes according to heart failure hospitalization history

**Figure 2:**
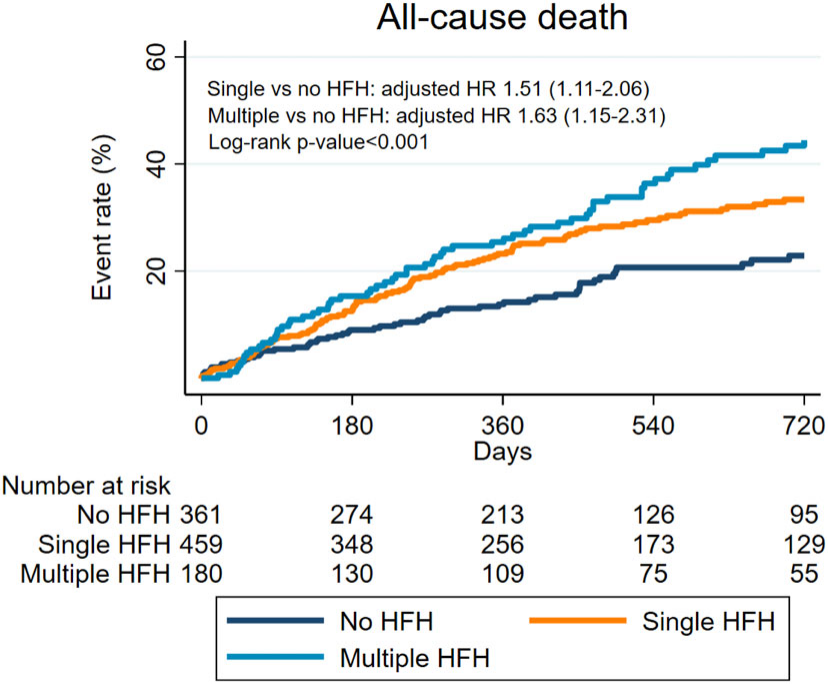
Kaplan–Meier curves for all‐cause mortality at 2 years stratified by the number of heart failure hospitalizations (HFH) prior to tricuspid transcatheter edge‐to‐edge repair. HR, hazard ratio.

After a median follow‐up of 391 (181–746) days, 297 patients (30%) died and 418 (42%) experienced the composite endpoint of all‐cause death or HFH. Single and multiple HFHs before T‐TEER were associated with an increased risk of death at both univariable and multivariable analysis as compared to no HFH (single vs. no HFH, crude HR 1.60 [95% CI 1.20–2.12], *p* = 0.001; adjusted HR 1.51 [95% CI 1.11–2.06], *p* = 0.01; multiple vs. no HFH, crude HR 2.15 [95% CI 1.57–2.96], *p* < 0.001; adjusted HR 1.63 [95% CI 1.15–2.31], *p* = 0.006) (*Figure* *2*).

**Figure 3:**
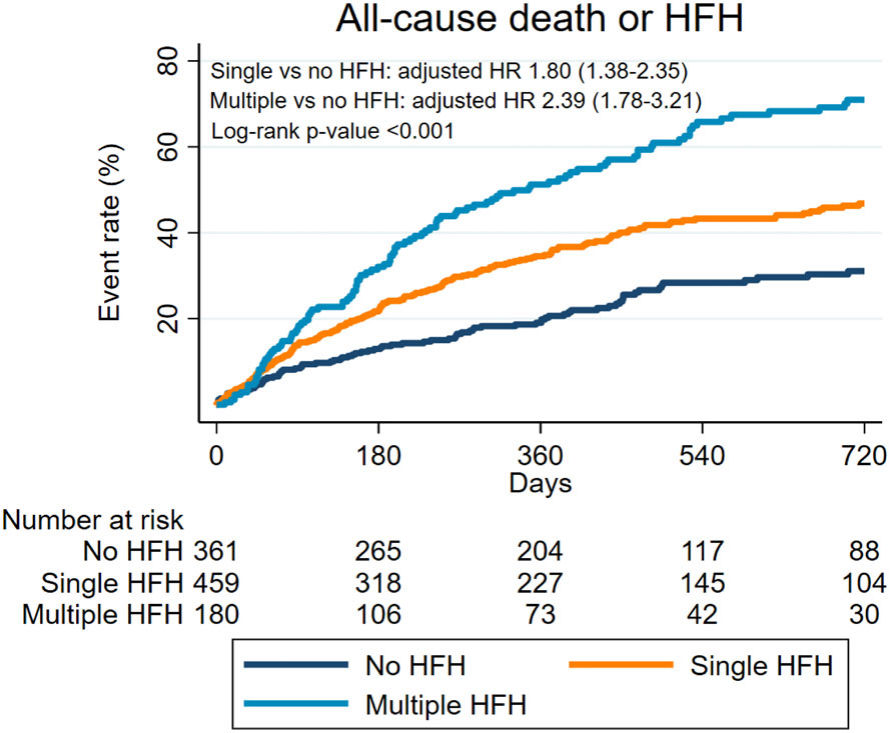
Kaplan–Meier curves for the combined endpoint of all‐cause death or heart failure hospitalization (HFH) at 2 years, stratified by the number of HFHs prior to tricuspid transcatheter edge‐to‐edge repair. HR, hazard ratio.

Single and multiple HFHs were also associated with a higher risk of the composite endpoint of all‐cause mortality or HFH (single vs. no HFH, crude HR 1.82 [95% CI 1.42–2.32], adjusted HR 1.80 [95% CI 1.38–2.35]; multiple vs. no HFH, crude HR 3.06 [95% CI 2.34–4.01], adjusted HR 2.39 [95% CI 1.78–3.21]; all *p* < 0.001) (*Figure* *3*).

Multiple HFHs did not further increase the risk of death as compared to single HFH (multiple vs. single HFH: crude HR 1.32 [95% CI 0.97–1.79]; *p* = 0.074; adjusted HR 1.12 [95% CI 0.83–1.51]; *p* = 0.466), but increased the risk of the combined endpoint (multiple vs. single HFH: crude HR 1.76 [95% CI 1.38–2.24], *p* < 0.001; adjusted HR 1.36 [95% CI 1.06–1.76], *p* = 0.016) (*Figures* *2* and *3*).

The association of HFH before T‐TEER with the risk of all‐cause death and of the combined endpoint was confirmed in sensitivity models including additional variables potentially associated with outcomes (e.g. coaptation gap, diuretic therapy, guideline‐directed medical therapy [GDMT] for HF) (online supplementary *Table* *S3*). Furthermore, the association of HFH before T‐TEER with the risk of all‐cause death and of the combined endpoint was confirmed after the exclusion of patients undergoing M‐TEER (online supplementary *Table* *S4*, *Figure* *S1*).

### Post‐procedural heart failure hospitalization and heart failure hospitalization trajectories

**Figure 4:**
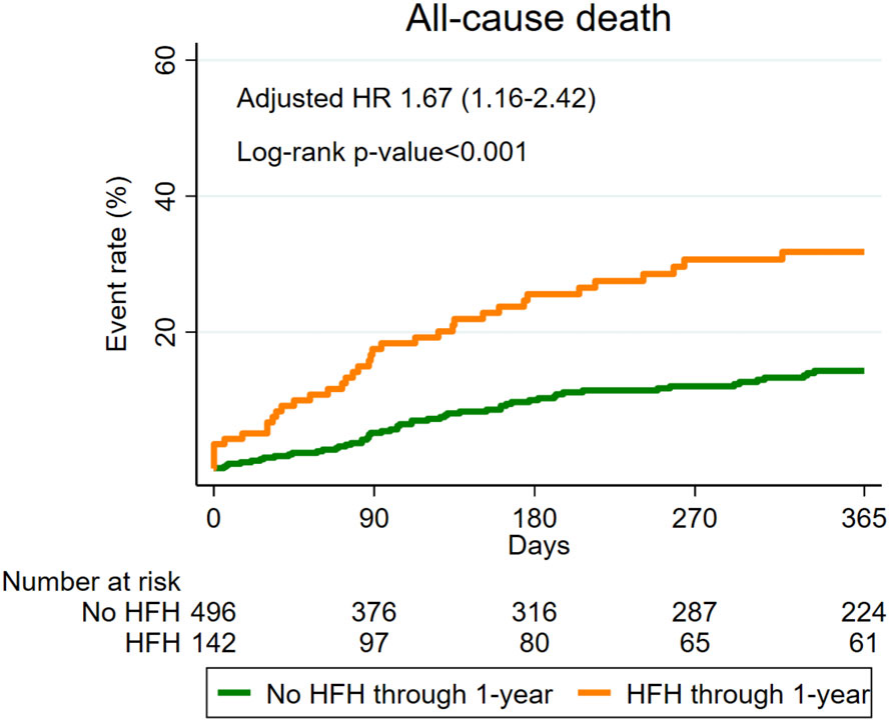
Subsequent incidence of death in patients with heart failure hospitalization (HFH) through 1 year after tricuspid transcatheter edge‐to‐edge repair. HR, hazard ratio.

Of the 638 patients treated with T‐TEER with at least 1‐year follow‐up, 142 (22%) were admitted at least once for HF in the year following the procedure. Post‐procedural HFH was associated with a higher risk of death (crude HR 1.95 [95% CI 1.40–2.71], *p* < 0.001; adjusted HR 1.67 [95% CI 1.16–2.42], *p* = 0.006) (*Figure* *4*). Data were consistent after the exclusion of patients undergoing M‐TEER (crude HR 2.27 [95% CI 1.52–3.37], *p* < 0.001; adjusted HR 2.03 [95% CI 1.29–3.20], *p* = 0.002, *n* = 518) (online supplementary *Figure* *S2*).

**Figure 5:**
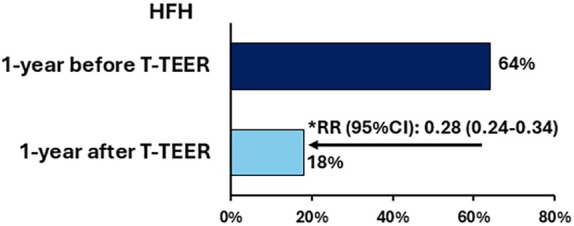
Heart failure hospitalization (HFH) during the 1 year before and after tricuspid transcatheter edge‐to‐edge repair (T‐TEER) in the EuroTR cohort and the relative risk ratio (RR). Risk of HFH was reduced by 72% in the year after T‐TEER compared with 1 year prior to the T‐TEER procedure. CI, confidence interval. *Calculated after excluding patients who died during the 1 year after T‐TEER and of patients without at least 1‐year follow‐up.

The probability of HFH was significantly reduced in the year after T‐TEER compared with 1 year prior to the T‐TEER procedure (RR 0.28 [95% CI 0.24–0.34], *p* < 0.001) (*Figure* *5*). The result was confirmed after excluding those undergoing M‐TEER during the study period (RR 0.35 [95% CI 0.30–0.42], *p* < 0.001).

**Figure 6:**
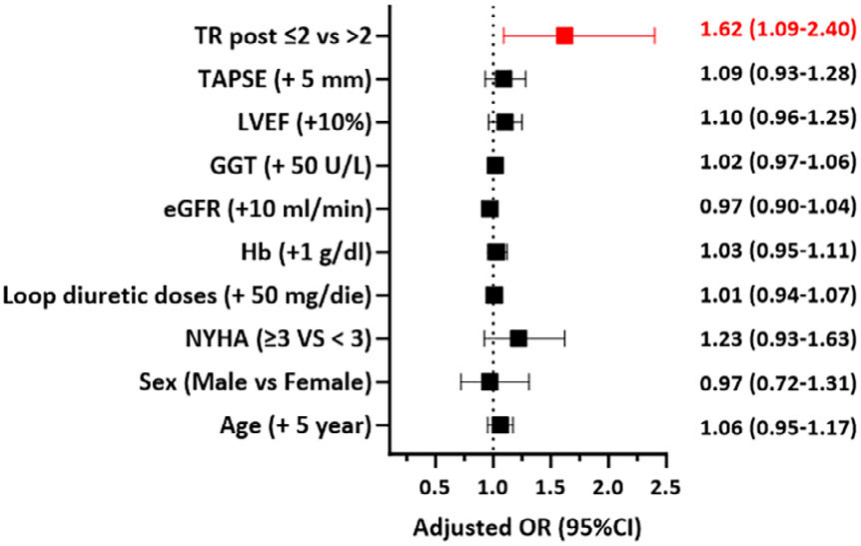
Predictors of improvement in the need of heart failure hospitalization (forest plot) – residual tricuspid regurgitation (TR) post‐tricuspid transcatheter edge‐to‐edge repair was the only independent predictor of improvement in clinical status. CI, confidence interval; eGFR, estimated glomerular filtration rate; GGT, gamma‐glutamyl transferase; Hb, haemoglobin; LVEF, left ventricular ejection fraction; NYHA, New York Heart Association; OR, odds ratio; TAPSE, tricuspid annular plane systolic excursion.

After T‐TEER, 20% of patients experienced worsening in HFH status, 39% were stable and 41% improved. Post‐procedural TR ≤2 was the only independent predictor of improvement in HFH status (odds ratio 1.62 [95% CI 1.09–2.40], *p* = 0.017) (*Figure* *6*). No significant differences were found in the rate of hospitalizations after T‐TEER between the subgroups of patients with residual TR 1+ versus 2+. Online supplementary *Figure* *S3* depicts the results of the sensitivity analysis.

## Discussion

The main findings of this study are as follows: (i) in a large, real‐word cohort of patients undergoing T‐TEER for symptomatic TR, approximately two‐thirds experienced at least one HFH in the year before the procedure; (ii) a history of HFH serves as a marker of more advanced HF and is associated with an increased risk of clinical events after T‐TEER, even after adjustment; (iii) in high‐risk patients from the EuroTR cohort, the relative risk of HFH was reduced by 72% in the year following T‐TEER compared to the year prior; and (iv) successful TR reduction (i.e. residual TR grade ≤2) was the only independent predictor of improvement in HFH status after T‐TEER.

### History of heart failure hospitalization

Limited evidence exists regarding the prevalence of HFH history in T‐TEER cohorts, with reported rates varying significantly based on patient characteristics. Notably, no studies have previously addressed the number of HFHs prior to tricuspid interventions. In the real‐world TriValve registry, the prevalence of prior HFH was 74.2%.^23^ Similarly, another real‐world cohort reported a 64.8% prevalence of HFH.^24^ In contrast, randomized trials and prospective registries, such as TRILUMINATE Pivotal (25.1%),^20^ bRIGHT (Observational Real‐World Study Evaluating Severe Tricuspid Regurgitation Patients Treated With the Abbott TriClip Device; 40%),^25^ TRISCEND (Tricuspid Valve Replacement: Investigation of Safety and Clinical Efficacy after Replacement of Tricuspid Valve with Transcatheter Device) early feasibility study (40.8%),^26^ TRISCEND II (31.4%),^27^ and Tri.Fr (40.3%)^28^ have shown lower rates. In EuroTR, approximately two‐thirds of patients had at least one HFH in the year before T‐TEER, with 45.9% having a single HFH and 18.0% multiple events.

Differences in HFH prevalence reflect varying patient profiles and may explain different outcomes. For example, the low HFH rate in TRILUMINATE Pivotal may reflect a lower‐risk population, contributing to the absence of significant HFH or mortality benefits in the device arm.^29^ EuroTR, as a prospective observational registry, cannot demonstrate procedure efficacy but provides valuable data on HFH prevalence and changes post T‐TEER in a real‐world setting. The findings of this study indicate that prior HFH is a strong, independent predictor of post‐procedural HFH and all‐cause mortality after T‐TEER, underscoring the need for high‐risk patient inclusion in future trials, such as the ongoing TRICuspid Intervention in Heart Failure (TRICI‐HF, NCT04634266).^29^ Including prior HFH as an eligibility criterion may help capture more hard endpoints (i.e. HFH or death) during the follow‐up.^29^

### Heart failure hospitalization history and outcomes

A key finding of this study is the association between HFH history and outcomes in patients undergoing T‐TEER. We found that a history of at least one HFH prior to T‐TEER is associated with a greater than 50% increased mortality risk post‐procedure. Additionally, multiple HFHs are associated with a greater risk of post‐procedural all‐cause death or HFH as compared with single HFH (HR 2.4 vs. 1.8). The prognostic impact of HFH in patients undergoing T‐TEER has never been reported. However, our results are in line with those reported for patients with left‐sided HF. In previous studies, patients with HF and prior HFH had more advanced symptoms, required higher doses of diuretics, and finally experienced up to three‐fold independent risk of mortality as compared with those not hospitalized.^1^, ^3^, ^4^, ^5^, ^6^ Also, multiple HFHs are considered as a marker of advanced HF carrying a poor prognosis in populations with HF.^30^, ^31^ Finally, similarly to our study, in a cohort of patients with advanced HF, >1 HFH in the last year was not associated with a higher risk of all‐cause death alone but was significantly associated with the risk of the composite endpoint of all‐cause death or HFH.^30^ The progressive increase in the risk of recurrent HFH events may clearly describe the trajectory of worsening HF.^1^

### Heart failure hospitalization trajectories

Data on HFH reduction after T‐TEER are scarce. The TRILUMINATE Pivotal trial demonstrated that T‐TEER was effective in reducing TR severity and improving quality of life, but with no significant between‐group differences in mortality or HFH at 1 year.^20^ Similarly, the Tri.Fr study, that randomized patients with ≥ severe symptomatic TR to T‐TEER with TriClip and GDMT or GDMT alone, met its primary endpoint, the Packer composite clinical endpoint evaluated at 12‐month post‐randomization, combining occurrence of major CV events (CV hospitalization and/or death), changes in NYHA class, or patient global assessment. Despite T‐TEER improved safely and significantly the composite clinical endpoint over a period of 12 months after randomization, statistical significance was not reached for major CV events nor for CV death.^28^

However, additional follow‐up might be necessary to determine prolonged benefits of T‐TEER in low‐risk patients. Two‐year outcomes of the TRILUMINATE Pivotal trial found that the annualized rate of recurrent HFH was significantly lower with T‐TEER compared with control (0.19 vs. 0.26 events/patient/year, *p* = 0.02; joint frailty model HR 0.72, one‐sided upper confidence limit of 0.93, *p* = 0.02).^32^

In the prospective, single‐arm, multicentre TRILUMINATE trial, a 49% reduction in all‐cause hospitalization, driven by an 84% reduction in HFH, was reported at 2‐year follow‐up.^33^ Among 119 patients undergoing isolated T‐TEER procedures for severe TR within compassionate use programmes in four academic centres, the estimated annual rate of HHF after T‐TEER was significantly reduced from 1.21 (95% CI 1.00–1.41) HFH/patient‐year before to 0.95 (95% CI 0.56–1.35) HFH/patient‐year after T‐TEER (*p* = 0.02), resulting in a 22% reduction in the annual rate of HFH.^34^ In the CLASP‐TR registry, a 56% reduction in the rate of HFH was observed at 1‐year follow‐up as compared to the year before T‐TEER.^35^

In our high‐risk population, the risk of HFH was significantly reduced by 72% during the 1 year after T‐TEER, as compared with 1 year before. Importantly, after the exclusion of patients undergoing M‐TEER for haemodynamic significant mitral regurgitation within the study period, the results were confirmed with a 65% reduction in the relative risk of HFH.

Although the retrospective nature and the lack of a conservatively treated control group cannot prove a causal benefit of T‐TEER treatment, our finding may support the hypothesis that treating the precipitant factor leading to HFH (i.e. TR) may reduce the risk of worsening HF itself. This is particularly true in patients at high risk for worsening HF (i.e. those with prior HFH).

### Predictors of improvement in heart failure hospitalization status

Currently, there is limited evidence on predictors of reduction in HFH after T‐TEER. In EuroTR, the only predictor of improvement in HFH status, meaning reduction in HFH after T‐TEER without dying, was residual TR grade ≤2.^36^ The association between residual TR after T‐TEER and prognosis has already been described and highlights the importance of patient selection for T‐TEER. In a previous analysis we showed that, while there were no differences in the 2‐year survival for residual TR 1+ versus 2+, residual TR ≥3+ was associated with worse outcomes.^37^ Similarly, we found no significant differences in HFH rates after T‐TEER between patients with mild versus moderate residual TR. Our finding highlights the potential role of the T‐TEER procedure in interrupting the progressive decline in the trajectory depicting the clinical status of patients with symptomatic TR (*Graphical Abstract*). Data from randomized controlled trials are needed to confirm our results.

### Limitations

EuroTR is an observational study. Therefore, despite the extensive adjustments, the role of residual unmeasured confounders cannot be ruled out. About 50% of patients enrolled in the EuroTR were excluded from the present analysis due to missing data on the number of HFHs which also limits the interpretability of the results. Importantly, the 1‐year mortality rate among excluded patients seems to be slightly lower as compared with the included population. Excluded patients might have a risk of morality that is similar to those without HFH in the previous year. This does not affect the results of our analysis, or rather strengthens their value, showing a benefit of T‐TEER in a high‐risk population with a high number of HFHs before T‐TEER.

Recurrent HFHs during follow‐up were not reported, preventing the analysis of recurrent HFH events.

Clinical endpoints were not adjudicated by an external, blinded, and unbiased clinical adjudication committee. Also, the strength of our findings is limited by the lack of a control group, that prevents to draw strong conclusions. Thus, our results should be considered as hypothesis‐generating only.

## Conclusions

A history of HFH was common and associated with an increased risk of HF readmission and mortality in patients with symptomatic TR undergoing T‐TEER. A significant reduction in HFHs was observed in the year following T‐TEER compared with the year preceding the procedure in high‐risk patients from the EuroTR cohort. Residual TR grade ≤2 emerged as the only independent predictor of improvement in HFH status post T‐TEER.

### PubMed Investigator List

Edoardo Pancaldi; Luca Branca; Ludwig T. Weckbach; Julia Novotny; Roman Pfister; Stephan Baldus; Muhammed Gerçek; Felix Rudolph; Sebastian Ludwig; Benedikt Koell; Leonhard‐Moritz Schneider; Dominik Felbel; Carsten Salomon; Harald Lapp; Tania Puscas; Alain Berrebi; Amir Abbas Mahabadi; Florian Schindhelm; Berenice Caneiro‐Queija; Julio C. Echarte; Jürgen Schreieck; Andreas Goldschmied; Natacha Rousse; Samy Aghezzaf; Norbert Frey; Martin J. Kraus; Leonie Ziegler; Sebastian Rosch; Federico Arturi; Andrea Panza; Matteo Mazzola; Cristina Giannini.

### Funding

Among the full cohort of patients in the registry, data collection for the Hamburg patients was supported by a grant from the German Heart Foundation.

**Conflict of interest**: M.A. received consulting fees in the last 3 years from Abbott Structural Heart and Edwards Lifesciences. J.H. reports research grant support and speaker honoraria from Edwards Lifesciences. K.P.K. is a consultant to Edwards Lifesciences and ReCor Medical. W.R. received speaker honoraria from Edwards and Abbott. P.D. served as consultant for InnovHeart, Picardia, HVR, Approxima, and received speaker honoraria from Abbott and Edwards. T.R. received speaker honoraria and consulting fees from AstraZeneca, Bayer, Pfizer, Daiichi Sankyo. M.B.P. received speaker fees from Abbott Vascular, Edwards Lifesciences and Venus Medtech. R.S.v.B. has received institutional grants and served as speaker to Abbott Vascular and Edwards Lifesciences. S.T. has received personal honoraria from Medtronic, Boston Scientific, Biosensors, Abbott Vascular, Medira, Shockwave, Teleflex, atHeart Medical, Cardiac Dimensions, Polares Medical, Amarin, Sanofi, AstraZeneca, ReCor Medical, Daiichi Sankyo, has received institutional research grants from Edwards Lifesciences, Boston Scientific, Fumedica, Novartis, Boehringer Ingelheim, and holds equity in Hi‐D Imaging. G.T. received speaker fee for Abbott Vascular and Edwards Lifesciences. A.S. served on an advisory board for Edwards Lifesciences. Tobias Geisler, received speaker honoraria/research grants from AstraZeneca, Bayer, Bristol Myers Squibb/Pfizer, Ferrer/Chiesi, Medtronic and Edwards Lifesciences. R.E.L. received speaker fees from Abbott Vascular, Edwards Lifesciences, Boston Scientific and Venus Medtech. P.L. received speaker honoraria and consulting fees from AstraZeneca, Bayer, Pfizer, Edwards Lifesciences, and research honoraria from Edwards Lifesciences. N.K. has received consultant fees from Edwards Lifesciences, Boston Scientific and Medtronic, and received proctor fees from Abbott. F.M. received grant and/or research institutional support from Abbott, Medtronic, Edwards Lifesciences, Biotronik, Boston Scientific Corporation, NVT, Terumo, Venus, and consulting fees, honoraria personal and institutional from Abbott, Medtronic, Edwards Lifesciences, Xeltis, Cardiovalve, Occlufit, Simulands, Mtex, Venus, Squadra, Valgen Royalty Income/IP Rights Edwards Lifesciences, and is shareholder (including share options) of Magenta, Transseptalsolutions, 4Tech. F.P. received travel expenses from Edwards Lifesciences, Abbott Vascular, Polares Medical, Medira, and Siemens Healthineers. M.K. received speaker honoraria from Edwards and Abbott. D.K. has received personal fees from Abbott Medical, Edwards Lifesciences, Pi‐Cardia Ltd and Medtronic Inc. V.R. received research grants from Abbott Medical, Boston Scientific, and Edwards Lifesciences. C.I. received consultant fees and travel expenses from Abbott Medical and Edwards Lifesciences. P.L. received institutional grants from Edwards Lifesciences and honoraria from Innoventrics. L.S. received speaker honoraria from Edwards Lifesciences. M.M. received consulting fees in the last 3 years from Abbott Structural Heart, AstraZeneca, Bayer, Boehringer Ingelheim, Edwards Lifesciences, Roche Diagnostics. All other authors have nothing to disclose.

## Supporting information


**Appendix S1.** Supporting Information.
