# Supplementary material for: Heart failure hospitalizations and clinical outcomes in patients undergoing tricuspid transcatheter edge‐to‐edge repair: Insights from EuroTR
Source: Eur J Heart Fail. 2025 Jul 18;27(8):1559–69. doi: 10.1002/ejhf.3757 (PMC12482852; doi:10.1002/ejhf.3757)
Supplement: Supplementary file 1 — Appendix S1. Supporting Information. [file EJHF-27-1559-s001.docx]

**Supplemental Material**

**Supplemental Table 1. Baseline characteristics of the population included vs excluded.**

| Factor | Included  N=1000 | Excluded  N=1152 | p-value |
| --- | --- | --- | --- |
| **Clinical** |  |  |  |
| Age (years) | 80 (76, 83) | 80 (76, 83) | 0.43 |
| Female Sex | 512 (51.2%) | 627 (55.1%) | 0.072 |
| BMI (kg/m2) | 25 (22, 28) | 25 (23, 29) | 0.034 |
| Arterial Hypertension | 843 (84.3%) | 738 (77.4%) | <0.001 |
| Dyslipidemia | 377 (37.9%) | 564 (59.2%) | <0.001 |
| Diabetes Mellitus | 238 (23.8%) | 250 (26.3%) | 0.20 |
| Previous Myocardial Infarction | 113 (11.3%) | 119 (12.1%) | 0.56 |
| Corory Artery Disease | 482 (48.2%) | 399 (36.1%) | <0.001 |
| Peripheral Artery Diesaes | 105 (16.9%) | 22 (15.5%) | 0.68 |
| Previous Stroke/TIA | 115 (11.5%) | 57 (11.0%) | 0.76 |
| Atrial fibrillation/flutter | 917 (91.7%) | 990 (89.8%) | 0.13 |
| COPD | 177 (17.7%) | 175 (17.2%) | 0.76 |
| History of cardiac surgery | 250 (25.0%) | 323 (31.2%) | 0.002 |
| Prior TV surgery | 8 (0.9%) | 9 (1.8%) | 0.18 |
| RV lead | 299 (29.9%) | 281 (24.5%) | 0.005 |
| NYHA   - I - II - III - IV | 8 (0.8%)  93 (9.3%)  730 (73.1%)  167 (16.7%) | 17 (1.5%)  205 (18.1%)  768 (67.7%)  145 (12.8%) | <0.001 |
| 6MWT (m) | 220 (150, 307) | 289 (195, 366) | <0.001 |
| TRI-SCORE | 6 (5, 8) | 6 (5, 8) | 0.74 |
| Heart Rate (bpm) | 72 (63, 82) | 76 (66, 87) | 0.052 |
| Peripheral edema | 660 (66.1%) | 433 (59.3%) | 0.004 |
| Ascites | 137 (13.7%) | 102 (14.0%) | 0.86 |
| Pleural Effusion | 231 (23.8%) | 130 (22.5%) | 0.57 |
| Any sign of RHF | 755 (76.6%) | 505 (69.0%) | <0.001 |
| Loop Diuretic | 926 (93.2%) | 904 (92.1%) | 0.31 |
| Furosemide-equivalent daily dose (mg) | 40 (20, 80) | 60 (30, 100) | <0.001 |
| Thiazide diuretic | 201 (20.2%) | 88 (22.3%) | 0.37 |
| MRA | 420 (42.1%) | 414 (42.3%) | 0.93 |
| Beta-blockers | 856 (85.9%) | 817 (83.2%) | 0.10 |
| RASI | 612 (61.4%) | 363 (56.8%) | 0.086 |
| SGLT2-i | 108 (11.4%) | 45 (35.7%) | <0.001 |
| **Laboratory** |  |  |  |
| Hemoglobin (g/dl) | 11.6 (10.3, 13.0) | 12.0 (10.6, 13.3) | <0.001 |
| Platelet count (*1000/l) | 185 (152, 234) | 183 (149, 231) | 0.65 |
| NTproBNP (pg/l) | 2728 (1498, 5254) | 2182 (1221, 4398) | <0.001 |
| Creatinine (mg/dl) | 1.33 (1.01, 1.80) | 1.30 (1.00, 1.75) | 0.082 |
| eGFR (ml/min) | 45 (32, 61) | 44 (32, 58) | 0.15 |
| Total bilirubin (mg/dl) | 0.82 (0.60, 1.20) | 0.79 (0.59, 1.14) | 0.17 |
| AST (U/l) | 29 (23, 36) | 28 (23, 35) | 0.96 |
| ALT (U/l) | 18 (13, 25) | 19 (13, 25) | 0.87 |
| GGT (U/l) | 93 (51, 178) | 100 (54, 189) | 0.097 |
| Alkaline Phosphatase (U/l) | 98 (72, 134) | 119 (90, 161) | <0.001 |
| INR | 1.28 (1.10, 1.62) | 1.15 (1.02, 1.40) | <0.001 |
| **Echocardiography** |  |  |  |
| LVEF (%) | 55 (46, 60) | 55 (47, 60) | 0.39 |
| LA volume (ml) | 101 (73, 141) | 82 (56, 120) | <0.001 |
| MR Severity   - No-trace - Mild - Moderate - Moderate-to-severe - Severe | 58 (5.9%)  544 (55.2%)  242 (24.6%)  242 (24.6%)  111 (11.3%) | 89 (8.7%)  663 (64.9%)  234 (22.9%)  28 (2.7%)  8 (0.8%) | <0.001 |
| RV basal EDD (mm) | 48 (43, 54) | 49 (43, 56) | 0.47 |
| RV mid EDD (mm) | 39 (34, 45) | 42 (36, 48) | <0.001 |
| RV EDA (cm2) | 25 (20, 32) | 26 (21, 33) | 0.023 |
| RV FAC | 38 (30, 43) | 42 (34, 50) | <0.001 |
| TAPSE (mm) | 17 (14, 20 | 17 (14, 20) | 0.83 |
| TR Severity   - Mild - Moderate - Severe - Massive - Torrential | 1 (0.1%)  29 (2.9%)  484 (48.6%)  293 (29.4%)  189 (19.0%) | 0 (0.0%)  27 (2.6%)  486 (45.8%)  355 (33.4%)  194 (18.3%) | 0.040 |
| TR etiology   - Primary - Secondary - Mixed | 48 (4.8%)  860 (86.8%)  83 (8.4%) | 76 (6.9%)  949 (85.7%)  82 (7.4%) | 0.12 |
| TR EROA (cm2) | 0.55 (0.40, 0.80) | 0.53 (0.40, 0.74) | 0.51 |
| TR Regurgitant Volume (ml) | 46 (34, 63) | 47 (36, 62) | 0.57 |
| Coaptation Gap (mm) | 5.4 (4.0, 7.2) | 6.0 (4.0, 8.0) | <0.001 |
| RA area (cm2) | 34 (28, 43) | 35 (28, 43) | 0.082 |
| Echo-PASP (mmHg) | 40 (31, 50) | 43 (35, 54) | <0.001 |
| **RHC** |  |  |  |
| RAP mean (mmHg) | 13 (9, 17) | 13 (10, 18) | 0.18 |
| RA V-wave (mmHg) | 17 (11, 23) | 15 (9, 17) | 0.028 |
| PAP systolic (mmHg) | 44 (35, 56) | 44 (35, 54) | 0.62 |
| PAP diastolic (mmHg) | 19 (14, 24) | 17 (13, 22) | <0.001 |
| PAP mean (mmHg) | 29 (23, 36) | 28 (23, 35) | 0.086 |
| PCWP mean (mmHg) | 19 (13, 24) | 18 (15, 23) | 0.84 |
| CO (l/min) | 3.93 (3.24, 5.07) | 3.60 (2.92, 4.12) | 0.025 |
| PVR | 2.50 (1.72, 3.75) | 2.81 (1.90, 4.12) | 0.35 |

Categorical variables are presented with N and percentage, continuous variables are presented with median (first quartile - third quartile range).

6MWT, 6-Minute Walking Test; ALT, alanine transaminase; AST, aspartate transaminase; BMI, Body Mass Index; CO, Cardiac Output; COPD, Chronic Obstructive Pulmonary Disease; EDA, End Diastolic Area; EDD, End Diastolic Diameter; eGFR, Estimated Glomerular Filtration Rate; ESA, End Systolic Area; EROA, Effective Regurgitant Orifice Area; EuroScore II, European System for Cardiac Operative Risk Evaluation; FAC, Fractional Area Change; GGT, gamma-glutamyl transferase; HFH, heart failure hospitalization; INR, International Normalized Ratio; LVEF, Left Ventricular Ejection Fraction; MR, Mitral Regurgitation; MRA, mineralcorticoid receptor antagonists; NTproBNP, N-terminal pro–B-type natriuretic peptide; NYHA, New York Heart Association; PAP, Pulmonary Artery Pressure; PCWP, Pulmonary Capillary Wedge Pressure; PASP, Pulmonary Artery Systolic Pressure; PVR, Pulmonary Vascular Resistance; RAP, Right Atrial Pressure; RASI, Renin-angiotensin system inhibitors; RHC, right heart catheterization; RHF, right heart failure; RV, Right Ventricle; SGLT2-I, Sodium-Glucose Transport Protein 2 Inhibitors; STS Score; Society of Thoracic Surgeons; TAPSE, Tricuspid Annular Plane Systolic Excursion; TIA Transient Ischemic Attack; TR, Tricuspid Regurgitation; TV, Tricuspid Valve.

**Supplemental Table 2. Sensitivity analysis after the exclusion of patients undergoing M-TEER: baseline characteristics of the population and residual TR stratified by the history of HFH before T-TEER.**

| **Variable** | **No HFH**  **321 (38%)** | **Single HFH**  **379 (45%)** | **Multiple HFH**  **141 (17%)** | **p-value** |
| --- | --- | --- | --- | --- |
| **Clinical** |  |  |  |  |
| Age (years) | 80 (76, 83) | 81 (76, 84) | 78 (74, 82) | 0.007 |
| Female Sex | 165 (51.4%) | 205 (54.1%) | 59 (41.8%) | 0.045 |
| BMI (kg/m2) | 26 (23, 29) | 25 (22, 28) | 24 (22, 28) | 0.27 |
| Arterial Hypertension | 265 (82.6%) | 324 (85.5%) | 124 (87.9%) | 0.29 |
| Dyslipidemia | 117 (36.7%) | 140 (37.1%) | 63 (45.0%) | 0.20 |
| Diabetes Mellitus | 66 (20.6%) | 93 (24.5%) | 46 (32.6%) | 0.021 |
| Previous Myocardial Infarction | 34 (10.6%) | 35 (9.2%) | 19 (13.5%) | 0.37 |
| Corory Artery Disease | 155 (48.3%) | 178 (47.0%) | 72 (51.1%) | 0.71 |
| Peripheral Artery Diesaes | 32 (12.2%) | 46 (17.8%) | 11 (19.3%) | 0.14 |
| Previous Stroke/TIA | 32 (10.0%) | 48 (12.7%) | 15 (10.6%) | 0.51 |
| Atrial fibrillation/flutter | 299 (93.1%) | 343 (90.5%) | 132 (93.6%) | 0.33 |
| COPD | 42 (13.1%) | 77 (20.3%) | 34 (24.1%) | 0.006 |
| History of cardiac surgery | 87 (27.1%) | 88 (23.2%) | 45 (31.9%) | 0.12 |
| Prior TV surgery | 4 (1.7%) | 3 (0.9%) | 0 (0.0%) | 0.27 |
| RV lead | 96 (29.9%) | 113 (29.8%) | 48 (34.0%) | 0.62 |
| NYHA   - I - II - III - IV | 5 (1.6%)  47 (14.6%)  237 (73.8%)  32 (10.0%) | 3 (0.8%)  32 (8.5%)  274 (72.5%)  69 (18.3%) | 0 (0.0%)  7 (5.0%)  102 (72.9%)  31 (22.1%) | <0.001 |
| 6MWT (m) | 244 (175, 320) | 206 (134, 307) | 224 (134, 290) | 0.014 |
| TRI-SCORE | 6 (5, 7) | 6 (5, 7) | 7 (6, 8) | <0.001 |
| Heart Rate (bpm) | 70 (62, 79) | 72 (63, 81) | 73 (66, 83) | 0.15 |
| Peripheral edema | 166 (51.7%) | 266 (70.4%) | 114 (80.9%) | <0.001 |
| Ascites | 13 (4.1%) | 58 (15.3%) | 36 (25.5%) | <0.001 |
| Pleural Effusion | 35 (10.9%) | 79 (21.2%) | 58 (41.7%) | <0.001 |
| Any sign of RHF | 205 (65.7%) | 297 (79.4%) | 123 (87.2%) | <0.001 |
| Loop Diuretic | 289 (90.9%) | 356 (94.4%) | 134 (95.7%) | 0.080 |
| Furosemide-equivalent daily dose (mg) | 40 (20, 80) | 40 (20, 80) | 60 (40, 125) | <0.001 |
| Thiazide diuretic | 46 (14.4%) | 87 (23.0%) | 31 (22.1%) | 0.012 |
| MRA | 125 (38.9%) | 163 (43.1%) | 70 (50.0%) | 0.085 |
| Beta-blockers | 262 (81.9%) | 329 (87.0%) | 120 (85.7%) | 0.16 |
| RASI | 193 (60.1%) | 227 (60.2%) | 85 (61.2%) | 0.98 |
| SGLT2-i | 35 (11.3%) | 53 (14.4%) | 19 (15.4%) | 0.36 |
| **Laboratory** |  |  |  |  |
| Hemoglobin (g/dl) | 12.1 (10.8, 13.3) | 11.5 (10.1, 12.9) | 11.1 (9.6, 12.4) | <0.001 |
| Platelet count (*1000/l) | 190 (151, 230) | 186 (148, 234) | 185 (157, 277) | 0.78 |
| NTproBNP (pg/l) | 2062 (1131, 3973) | 2874 (1721, 5344) | 3003 (1664, 6823) | <0.001 |
| Creatinine (mg/dl) | 1 (1, 2) | 1 (1, 2) | 1 (1, 2) | <0.001 |
| eGFR (ml/min) | 50 (33, 68) | 44 (33, 58) | 43 (27, 55) | <0.001 |
| Total bilirubin (mg/dl) | 1 (1, 1) | 1 (1, 1) | 1 (1, 1) | 0.88 |
| AST (U/l) | 29 (24, 36) | 28 (23, 36) | 27 (22, 36) | 0.77 |
| ALT (U/l) | 19 (14, 26) | 18 (13, 24) | 17 (13, 24) | 0.19 |
| GGT (U/l) | 82 (44, 164) | 94 (51, 176) | 120 (57, 220) | 0.007 |
| Alkaline Phosphatase (U/l) | 96 (71, 131) | 101 (78, 138) | 104 (76, 129) | 0.58 |
| INR | 1.20 (1.10, 1.56) | 1.30 (1.10, 1.70) | 1.29 (1.10, 1.68) | 0.21 |
| **Echocardiography** |  |  |  |  |
| LVEF (%) | 55 (47, 60) | 55 (49, 60) | 53 (45, 60) | 0.14 |
| LA volume (ml) | 90 (64, 122) | 99 (73, 142) | 110 (87, 158) | <0.001 |
| MR Severity   - No-trace - Mild - Moderate - Moderate-to-severe - Severe | 22 (7.0%)  193 (61.1%)  98 (31.0%)  3 (0.9%)  0 (0.0%) | 25 (6.8%)  240 (65.0%)  93 (25.2%)  11 (3.0%)  0 (0.0%) | 11 (7.8%)  100 (70.9%)  24 (17.0%)  6 (4.3%)  0 (0.0%) | 0.040 |
| RV basal EDD (mm) | 47 (42, 54) | 48 (43, 54) | 50 (43, 55) | 0.12 |
| RV mid EDD (mm) | 38 (32, 44) | 40 (34, 45) | 41 (36, 47) | <0.001 |
| RV EDA (cm2) | 23 (19, 30) | 25 (20, 32) | 29 (25, 34) | <0.001 |
| RV FAC | 38 (30, 44) | 38 (30, 45) | 37 (29, 42) | 0.35 |
| TAPSE (mm) | 17 (14, 20) | 17 (14, 20) | 16 (13, 19) | 0.22 |
| TR Severity   - Mild - Moderate - Severe - Massive   Torrential | 1 (0.3%)  5 (1.6%)  143 (45.0%)  97 (30.5%)  72 (22.6%) | 0 (0.0%)  9 (2.4%)  179 (47.4%)  114 (30.2%)  76 (20.1%) | 0 (0.0%)  8 (5.7%)  63 (44.7%)  40 (28.4%)  30 (21.3%) | 0.34 |
| TR etiology   - Primary - Secondary   Mixed | 10 (3.2%)  281 (88.9%)  25 (7.9%) | 18 (4.8%)  324 (86.4%)  33 (8.8%) | 13 (9.2%)  114 (80.9%)  14 (9.9%) | 0.075 |
| TR EROA (cm2) | 0.60 (0.42, 0.88) | 0.60 (0.41, 0.81) | 0.52 (0.38, 0.77) | 0.036 |
| TR Regurgitant Volume (ml) | 48 (36, 66) | 47 (37, 67) | 43 (31, 60) | 0.016 |
| Coaptation Gap (mm) | 6 (4, 8) | 6 (4, 8) | 6 (4, 7) | 0.81 |
| RA area (cm2) | 32 (27, 41) | 35 (28, 44) | 35 (29, 42) | 0.065 |
| Echo-PASP (mmHg) | 36 (30, 48) | 40 (31, 50) | 42 (33, 49) | 0.017 |
| **RHC** |  |  |  |  |
| RAP mean (mmHg) | 12 (8, 16) | 12 (8, 16) | 12 (9, 16) | 0.89 |
| RA V-wave (mmHg) | 16 (11, 22) | 17 (11, 23) | 18 (12, 23) | 0.52 |
| PAP systolic (mmHg) | 41 (33, 51) | 44 (36, 55) | 49 (41, 60) | <0.001 |
| PAP diastolic (mmHg) | 17 (13, 22) | 18 (14, 23) | 22 (17, 28) | <0.001 |
| PAP mean (mmHg) | 27 (21, 33) | 30 (23, 36) | 32 (26, 39) | <0.001 |
| PCWP mean (mmHg) | 17 (12, 22) | 18 (14, 24) | 21 (14, 26) | 0.002 |
| CO (l/min) | 3.90 (3.20, 4.80) | 3.79 (3.21, 5.09 | 4.20 (3.50, 5.53) | 0.011 |
| PVR | 2.43 (1.72, 3.60) | 2.50 (1.66, 3.74) | 2.29 (1.70, 3.57) | 0.93 |
| **Residual TR at discharge**   - None - Mild - Moderate - Severe - Massive - Torrential | 28 (8.8%)  133 (41.6%)  98 (30.6%)  49 (15.3%)  9 (2.8%)  3 (0.9%) | 23 (6.1%)  153 (40.6%)  128 (34.0%)  56 (14.9%)  11 (2.9%)  6 (1.6%) | 5 (3.5%)  60 (42.6%)  50 (35.5%)  23 (16.3%)  3 (2.1%)  0 (0.0%) | 0.61 |

Categorical variables are presented with N and percentage, continuous variables are presented with median (first quartile - third quartile range).

6MWT, 6-Minute Walking Test; ALT, alanine transaminase; AST, aspartate transaminase; BMI, Body Mass Index; CO, Cardiac Output; COPD, Chronic Obstructive Pulmonary Disease; EDA, End Diastolic Area; EDD, End Diastolic Diameter; eGFR, Estimated Glomerular Filtration Rate; ESA, End Systolic Area; EROA, Effective Regurgitant Orifice Area; EuroScore II, European System for Cardiac Operative Risk Evaluation; FAC, Fractional Area Change; GGT, gamma-glutamyl transferase; HFH, heart failure hospitalization; INR, International Normalized Ratio; LVEF, Left Ventricular Ejection Fraction; MR, Mitral Regurgitation; MRA, mineralcorticoid receptor antagonists; NTproBNP, N-terminal pro–B-type natriuretic peptide; NYHA, New York Heart Association; PAP, Pulmonary Artery Pressure; PCWP, Pulmonary Capillary Wedge Pressure; PASP, Pulmonary Artery Systolic Pressure; PVR, Pulmonary Vascular Resistance; RAP, Right Atrial Pressure; RASI, Renin-angiotensin system inhibitors; RHC, right heart catheterization; RHF, right heart failure; RV, Right Ventricle; SGLT2-I, Sodium-Glucose Transport Protein 2 Inhibitors; STS Score; Society of Thoracic Surgeons; TAPSE, Tricuspid Annular Plane Systolic Excursion; TIA Transient Ischemic Attack; TR, Tricuspid Regurgitation; TV, Tricuspid Valve.

**Supplemental Table 3. Multivariable Cox model for all-cause death and for the combined endpoint after the inclusion of additional variables potentially associated with outcome.**

| **+ Coaptation gap** |  |  |
| --- | --- | --- |
| **Variable** | **Adjusted HR (95%CI)** | **p-value** |
| **All-cause death** |  |  |
| Single HFH | 1.50 (1.03-2.17) | 0.034 |
| Multiple HFH | 1.68 (1.13-2.50) | 0.010 |
| **Combined endpoint** |  |  |
| Single HFH | 1.64 (1.21-2.24) | 0.002 |
| Multiple HFH | 2.28 (1.64-3.16) | < 0.001 |
| **+ RV-FAC** |  |  |
| **Variable** | **Adjusted HR (95%CI)** | **p-value** |
| **All-cause death** |  |  |
| Single HFH | 1.41 (1.03-1.93) | 0.031 |
| Multiple HFH | 1.40 (0.97-2.01) | 0.069 |
| **Combined endpoint** |  |  |
| Single HFH | 1.63 (1.25-2.14) | < 0.001 |
| Multiple HFH | 2.13 (1.57-2.89) | < 0.001 |
| **+ Diuretics and GDMT** |  |  |
| **All-cause death** |  |  |
| Single HFH | 1.50 (1.09-2.01) | 0.012 |
| Multiple HFH | 1.86 (1.28-2.69) | 0.001 |
| **Combined endpoint** |  |  |
| Single HFH | 1.80 (1.37-2.36) | < 0.001 |
| Multiple HFH | 2.79 (2.04-3.82) | < 0.001 |

**Supplemental Table 4. Sensitivity analysis after the exclusion of patients undergoing M-TEER: univariable and multivariable Cox model for all-cause death and for the combined endpoint.**

| **Variable** | **Crude HR (95%CI)** | **p-value** | **Adjusted HR (95%CI)** | **p-value** |
| --- | --- | --- | --- | --- |
| **All-cause death** | | | | |
| Single HFH | 1.55 (1.12-2.13) | 0.007 | 1.49 (1.05-2.11) | 0.027 |
| Multiple HFH | 2.24 (1.56-3.21) | <0.001 | 1.64 (1.10-2.46) | 0.016 |
| **Combined endpoint** | | | | |
| Single HFH | 1.80 (1.37-2.36) | <0.001 | 1.85 (1.38-2.49) | < 0.001 |
| Multiple HFH | 3.31 (2.45-4.48) | <0.001 | 2.55 (1.83-3.58) | < 0.001 |

HFH, heart failure hospitalization; HR, hazard ratio

**Supplemental Figure 1.** **Sensitivity analysis after the exclusion of patients undergoing M-TEER: Kaplan Meier curves for a) all-cause mortality and b) the combined endpoint of all-cause death or HF hospitalization at 2-years, stratified by the number of HF hospitalizations prior to T-TEER.**

**a)**


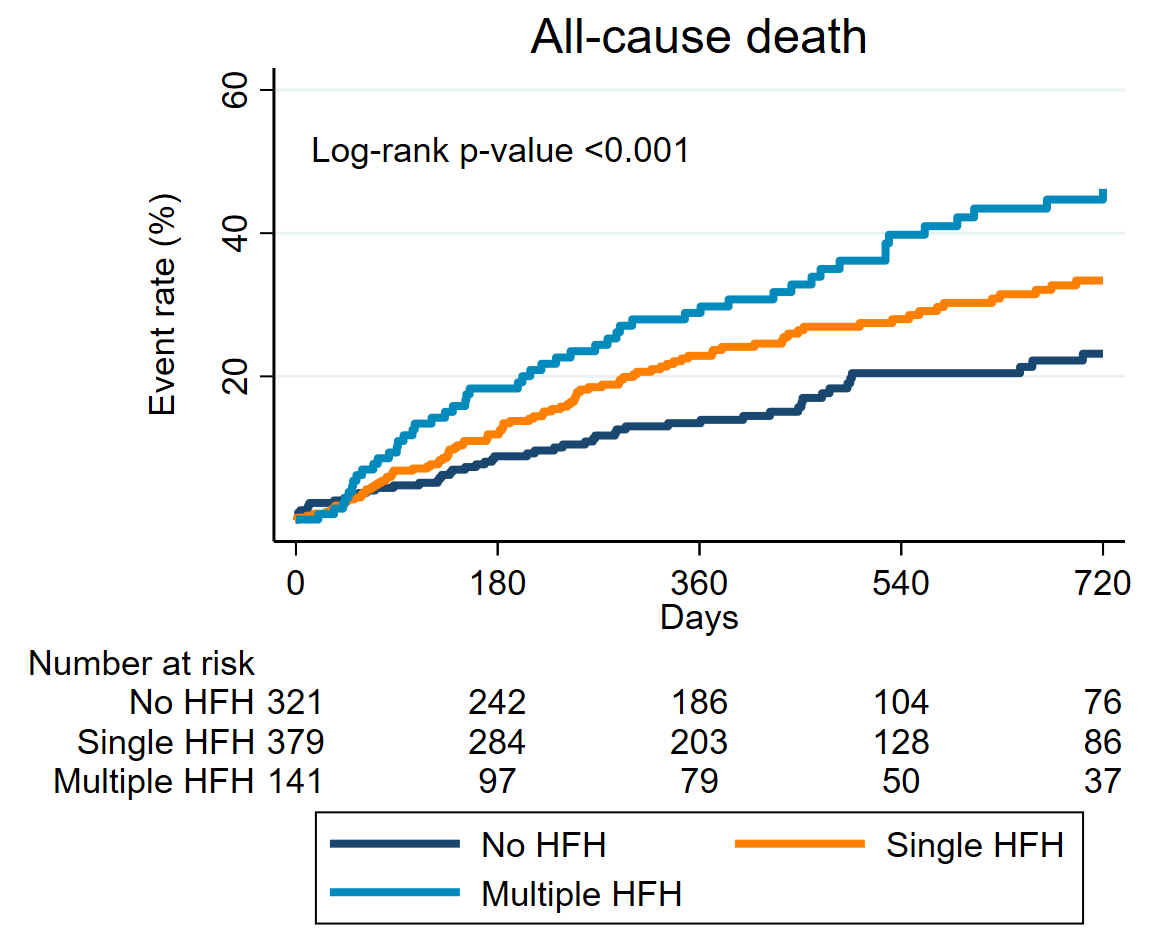


**b)**


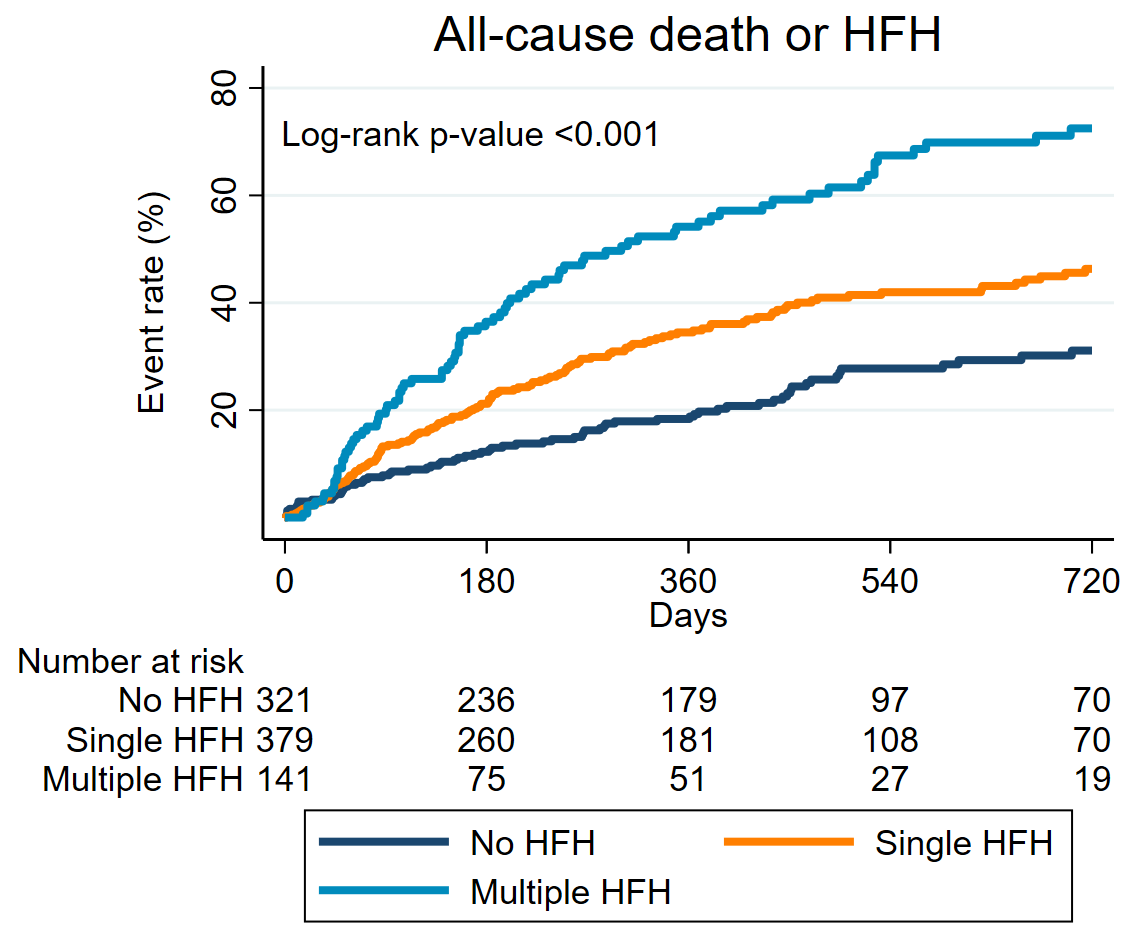


Abbreviations: HFH, heart failure hospitalization.

**Supplemental Figure 2.** **Sensitivity analysis after the exclusion of patients undergoing M-TEER: subsequent incidence of death in patients with HFH through 1 year after T-TEER (N=518).**


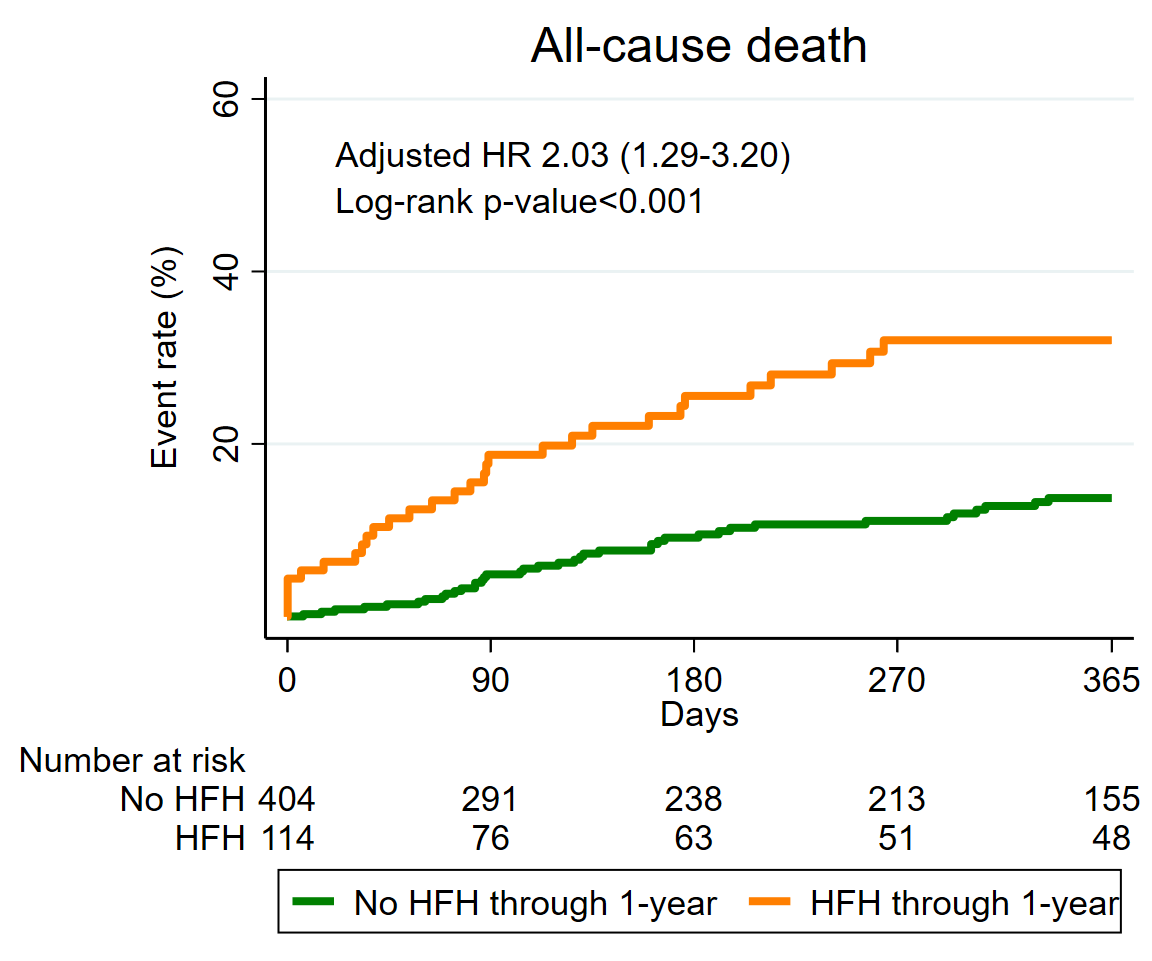


Abbreviations: HFH, heart failure hospitalization; HR, hazard ratio

**Supplemental Figure 3. Sensitivity analysis after the exclusion of patients undergoing M-TEER within the study period: trajectories in HFH status after T-TEER (left panel) and predictors of improvement (right panel).** **Residual TR post T-TEER was the only independent predictor of improvement in clinical status.**


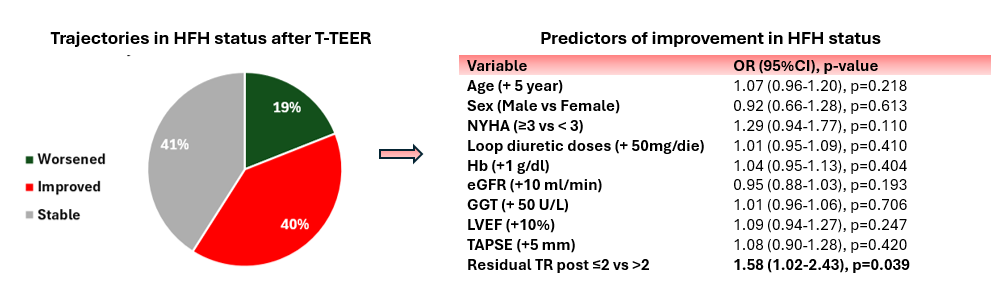


Abbreviations: eGFR, estimated glomerular filtration rate; GGT, gamma-glutamyl transferase; Hb, haemoglobin; HFH, heart failure hospitalization; LVEF, left ventricular ejection fraction; NYHA, New York Heart Association; TAPSE, tricuspid annular plane systolic excursion; TR, tricuspid regurgitation; T-TEER, tricuspid-transcatheter edge-to-edge repair.
